# DNA/RNA hybrids avoid channel gating that leads to the continued packaging of numerous hybrids into the phi29 protein shell

**DOI:** 10.1093/nar/gkaf242

**Published:** 2025-04-07

**Authors:** Dan Shu, Abhjeet S Bhullar, Chenxi Liang, Long Zhang, Peixuan Guo

**Affiliations:** Center for RNA Nanobiotechnology and Nanomedicine, The Ohio State University, Columbus, OH 43210, United States; Division of Pharmaceutics and Pharmacology, College of Pharmacy, The Ohio State University, Columbus, OH 43210, United States; Center for RNA Nanobiotechnology and Nanomedicine, The Ohio State University, Columbus, OH 43210, United States; Interdisciplinary Biophysics Graduate Program, College of Arts and Sciences, The Ohio State University, Columbus, OH 43210, United States; Center for RNA Nanobiotechnology and Nanomedicine, The Ohio State University, Columbus, OH 43210, United States; Division of Pharmaceutics and Pharmacology, College of Pharmacy, The Ohio State University, Columbus, OH 43210, United States; Center for RNA Nanobiotechnology and Nanomedicine, The Ohio State University, Columbus, OH 43210, United States; Division of Pharmaceutics and Pharmacology, College of Pharmacy, The Ohio State University, Columbus, OH 43210, United States; Center for RNA Nanobiotechnology and Nanomedicine, The Ohio State University, Columbus, OH 43210, United States; Division of Pharmaceutics and Pharmacology, College of Pharmacy, The Ohio State University, Columbus, OH 43210, United States; Interdisciplinary Biophysics Graduate Program, College of Arts and Sciences, The Ohio State University, Columbus, OH 43210, United States; College of Medicine, Dorothy M. Davis Heart and Lung Research Institute and James Comprehensive Cancer Center, The Ohio State University, Columbus, OH 43210, United States

## Abstract

Packaging of double-stranded DNA (dsDNA) into viral capsids is crucial in dsDNA viruses, including herpesviruses, adenoviruses, poxviruses, and bacteriophages. An ATPase motor compacts genomes. The phi29 DNA packaging motor, a model system, employs a hexameric pRNA (packaging RNA) ring and ATPase, sharing a revolving mechanism observed in herpesvirus genome packaging, bacterial DNA transport, Holliday junction resolution, and plasmid conjugation. Channel gating terminates translocation and readies a reversed pore for dsDNA exit; its mechanism is unclear. We report a packaging efficiency difference between dsDNA and RNA/DNA hybrids. Single-channel electrophysiology and sucrose gradient ultracentrifugation reveal that packaging fails if both ends are dsRNA, but succeeds if either 5′ or 3′ end is DNA. As long as one strand is DNA, RNA/DNA hybrids are packaged, with a higher copy number than dsDNA. Single-pore conductance assays show that this efficiency results from the absence of channel gating. The channel remains open during RNA/DNA translocation and does not close after hybrid packaging, implying dsDNA’s role in gating and conformational changes. This gating arises from dsDNA’s interaction with three flexible loops of the motor channel. These findings offer a structural and chemical foundation for designing containers to package RNA/DNA hybrids for gene/RNAi delivery, therapy, synthetic biology, nanotechnology, and single-particle sensing.

## Graphical Abstract

**Graphical Abstract:**
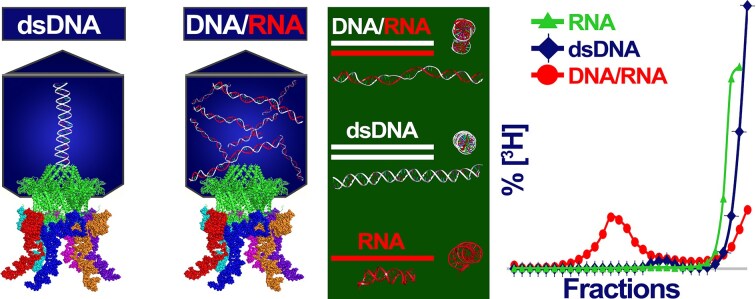

## Introduction

The assembly and packaging of double-stranded DNA (dsDNA) into viral capsids represent pivotal steps in the life cycle of many bacteriophages and other viruses [1–3]. These processes are driven by highly efficient, ATP-dependent molecular motors that overcome significant entropic and electrostatic barriers to compact large genomes into confined capsid spaces [4–10]. Among these, the bacteriophage phi29 DNA packaging motor has emerged as a well-characterized model, utilizing a hexameric pRNA (packaging RNA), a hexameric ATPase (gp16), and a dodecameric connector complex to translocate dsDNA into preformed procapsids [1, 4, 5, 8, 11–22]. Traditionally, the phi29 motor was believed to employ a rotational mechanism for DNA translocation. However, recent advances have shifted this paradigm, revealing that the motor instead uses a sequential revolving mechanism, mitigating torsional strain on the DNA helix [1, 6, 7, 10, 23]. The sequential action is a consequence of the conformational transition promoted by ATP binding and ATP hydrolysis. The revolving mechanism of the phi29 DNA packaging motor has been shown to share commonalities with the revolving mechanism for the transportation of the dsDNA of herpesvirus genome [24], bacterial genome [25], Holiday junction [26], and plasmid conjugation of *Actinomyces* [27]. This novel mechanism underscores the adaptability of the motor, enabling it to efficiently package long genomic dsDNA without inducing coiling or tangling.

Distinct nucleic acid substrates, such as RNA/DNA hybrids and dsRNA, exhibit varying structural properties that can significantly influence their interactions with viral packaging motors [28–30]. DNA/RNA hybrids, which assume an A-form helical structure, differ substantially from the canonical B-form structure of dsDNA in terms of helical pitch, groove dimensions, and rigidity [31–35]. Specifically, RNA/DNA hybrids feature a base rise of ∼0.26 nm and a helical pitch of 2.86 nm, whereas dsDNA has a base rise of 0.34 nm and a pitch of 3.36 nm [32]. Understanding how these structural differences affect the translocation efficiency and channel gating of the phi29 motor is crucial for elucidating the molecular mechanisms that underlie substrate selectivity.

Studying the viral DNA packaging process of the phi29 motor complex revealed that the channel pore undergoes three steps of gating, under which the channel pore diameter is reduced in the presence of a different electrical potential difference across the pore connector. Our study explores the differences in this gating behavior as a function of the composition of the translocating nucleic acid instead of a fixed potential difference [1, 3, 15, 36–40]. The gating is the conformational change related to the transition process for the motor to cover both the inward packaging of DNA entry into the capsid and the outward release of the dsDNA exiting during infection. The gating is a consequence of the interaction of dsDNA with the flexible channel loops inside the channel and at the C-terminal of the connector internal end point [1, 3, 36–40].

The conformation [8, 37] of the phi29 motor channel may respond to various nucleic acid substrates. The phi29 DNA packaging motor is among the most powerful biomotors characterized, capable of generating forces of up to 57 pN during DNA translocation [21, 41]. This remarkable force generation is facilitated by the coordinated action of its components, including the connector protein (gp10), which forms a dodecameric ring structure that serves as a conduit for DNA entry into the procapsid. Additionally, a unique pRNA forms a hexameric ring essential for efficient DNA translocation [1, 5, 8, 11, 17, 19, 39, 42, 43]. Understanding the interplay between substrate structure and motor function is crucial for advancing our knowledge of viral DNA packaging mechanisms, with significant implications for applications in the structures used for RNA nanotechnology generally and in gene therapy [1–3, 44–46]. The phi29 motor functions through a synergistic interaction of its core components: the connector protein, the pRNA ring, and the ATPase (gp16) [5, 8, 47–49]. The ATPase hydrolyzes ATP to generate the mechanical force required for DNA packaging, propelling nucleic acids at speeds reaching up to 100 bp/s [1, 4, 5, 8, 50]. This rapid translocation is further facilitated by the motor’s sequential revolving mechanism, ensuring unidirectional movement without introducing torsional stress [1, 10, 23, 51]. Recent studies indicate that the motor’s gating mechanism is influenced not only by the physical structure of the nucleic acid but also by its chemical properties [1, 15, 36].

RNA/DNA hybrids exhibit an A-form conformation with a deeper major groove and a shorter pitch than the B-form of dsDNA, which will reduce steric hindrance and electrostatic resistance within the motor channel [33, 34, 52–54]. How such structural differences influence packaging efficiency is an intriguing question. Furthermore, the positively charged lysine residues lining the motor channel interact strongly with the negatively charged phosphate backbone of dsDNA, creating a more constrained environment that might limit its translocation efficiency [1, 4]. The flexibility of RNA/DNA hybrids may alleviate these interactions, leading to smoother translocation. This flexibility aligns with findings from other ATP-driven motors, such as those in the T4 bacteriophage system, where substrate flexibility enhances packaging rates [29, 37, 55–63].

The goal of this investigation is to dissect the influence of substrate flexibility, electrostatic interactions, and sequence specificity on the phi29 DNA packaging motor. We observed discrepancy in how efficiently dsDNA and RNA/DNA hybrids are packaged. Surprisingly, RNA/DNA hybrids display a far greater packaging efficiency than dsDNA. Single-channel conductance experiments demonstrate that this enhanced efficiency stems from the absence of channel gating. Consequently, newly formed RNA/DNA hybrid complexes can continue translocating into the same procapsid uninterrupted until it becomes packed with more nucleic acids than dsDNA alone. The motor channel remains largely open during RNA/DNA hybrid insertion, implying that dsDNA itself governs the gating and structural adjustments of the DNA packaging motor. These findings establish both structural and chemical foundations for engineering containers capable of holding extensive quantities of RNA/DNA hybrids. Such containers could be employed for nucleic acid delivery in gene therapy or utilized in single-molecule sensing applications using labeled RNA/DNA hybrids.

## Materials and methods

### Design and preparation of nucleic acid substrates

Nonradioactive strands (Table 1) were sourced from Integrated DNA Technologies. For electrophoresis analysis, the (+) strand of dsDNA was labeled with Alexa 647 at its 5′ end. Radioactive dsDNA was synthesized using the polymerase chain reaction (PCR) method (see details below), while other double-stranded substrates were produced by annealing two complementary single strands using a thermocycler in TES buffer (10 mM Tris–HCl, pH 7.5, 1 mM EDTA, 50 mM NaCl). The annealing protocol involved heating to 95°C (for DNA) or 85°C (for RNA/DNA hybrids) for 5 min, followed by gradual cooling, reducing the temperature by 1°C every 30 s until reaching 4°C. Proper annealing of product was confirmed with gel electrophoresis (native gel 12% TBE) seen in Supplementary Fig. S1.

**Table 1.** Sequences of the nucleic acid substrate

Names	Sequences (5′–3′)
DNA1	GCTGGATGTCACCGGATTGTCGGACATCGGATTGTCTGAGTCATATGACACATCCAGC
DNA2	GCTGGATGTGTCATATGACTCAGACAATCCGATGTCCGACAATCCGGTGACATCCAGC
5′ PolyA DNA2	AAAAAAAAAAAAAAAAAAAAGCTGGATGTGTCATATGACTCAGACAATCCGATGTCCGACAATCCGGTGACATCCAGC
3′ PolyA DNA2	GCTGGATGTGTCATATGACTCAGACAATCCGATGTCCGACAATCCGGTGACATCCAGC AAAAAAAAAAAAAAAAAAAA
5′DNA3′RNA2	GCTGGATGTGTCATATGACTCAGACAATCCGATGTCCGAC*AAUCCGGUGACAUCCAGC*
5′RNA3′DNA2	*GCUGGAUGUGUCAUAUGA* CTCAGACAATCCGATGTCCGACAATCCGGTGACATCCAGC
RNA1	*GCUGGAUGUCACCGGAUUGUCGGACAUCGGAUUGUCUGAGUCAUAUGACACAUCCAGC*
RNA2	*GCUGGAUGUGUCAUAUGACUCAGACAAUCCGAUGUCCGACAAUCCGGUGACAUCCAGC*
RNA3	*GGAATGGUACGGUACUUCCAUUGUCAUGAAUAUCACUCUGAGACAACUUUUCUAUUGCGUGUCAAUCAUGGCCU*
RNA–DNA–RNA	*AGGCCAUGAUUGACACGCAAU* AGAAAAGTTGTCTCAGAGTGATATT*CAUGACAAUGGAAGUACCGUACCAUUCU*

### Radioactive labeling of nucleic acids

Radioactive labeling of the RNA strand within DNA/RNA hybrids was achieved using *in vitro* transcription via the MEGAscript™ T7 Transcription Kit (Thermo Fisher Scientific, Cat. AMB1334) supplemented with ^3^H-UTP (PerkinElmer, Cat. NET380). For each transcription reaction, 8 μl of ^3^H-UTP was incorporated, while the concentration of cold rUTP was decreased by a factor of 4. For radioactive dsDNA, labeling was performed using the PCR method with ^3^H-TTP (PerkinElmer, Cat. NET221). The reaction included 20 μl of ^3^H-TTP in a total volume of 100 μl, with the concentration of dTTP adjusted to one-fourth of its standard level.

### Ultracentrifugation assay for DNA packaging

The packaging reactions were prepared as described and incubated at room temperature for 1 h. Following the reaction, the mixture was directly layered onto a 5–20% sucrose gradient within a 5-ml open-top ultracentrifuge tube. Samples were centrifuged using an SW55 Ti swinging-bucket rotor at 35 000 rpm for 30 min at 20°C. Fractions of ∼150 μl were collected sequentially, and the tritium radioactivity in 100 μl from each fraction was quantified using a scintillation counter. To normalize data, the radioactivity or fraction number for each sample was divided by the sum of the total radioactivity of all fractions. The fraction number was also normalized relative to the highest fraction count.

### DNA packaging into viron

DNA packaging assay has been previously described [22]. The viral prohead protein complex and gp16 were cloned and expressed in *Escherichia coli* [22, 49]. pRNA was produced using MEGAscript™ T7 Transcription Kit (Thermo Fisher Scientific, Cat. AMB1334) [64]. pRNA, gp16, and 1 μl of 5 μM different substrate were mixed together, and then incubated at room temperature for 1 h.

### Insertion of nano-apertures into liposomes for channel gating analysis

The protocol for generating proteoliposomes containing protein apertures was adapted from previously established methods [1, 15, 36]. In brief, 1 mg of DPhPC in chloroform was evaporated using a rotary evaporator (∼5 min). The resulting lipid film was rehydrated with a buffer containing 3 M KCl, 250 mM sucrose, and 5 mM HEPES (pH 7.4), followed by vortexing to dissolve the lipids thoroughly. The protein concentration in the solution was adjusted to 200–500 μg/ml. To ensure uniform proteoliposome formation, the suspension was passed through a 0.4-μm polycarbonate membrane using an extruder (Avanti Polar Lipids) for 20–30 repetitions.

### Electrophysiology measurement for packaging and gating

Lipid bilayers were formed on a Teflon partition membrane with a pore size of 200 μm using an Axon Clamp setup. Ag/AgCl electrodes were positioned in both the *cis* and *trans* chambers. A Bilayer Clamp Amplifier BC-535 (Warner Instruments) was connected to an Axon DigiData 1440A analog–digital converter (Molecular Devices). Data acquisition was performed at a bandwidth of 1 kHz with a sampling frequency of 20 kHz. Clampex 10 and Clampfit 10 software (Molecular Devices) were employed for data collection and analysis.

### Analysis of single gating event using MinION™ for high-throughput analysis

The insertion of pores into the MinION™ flow cell was conducted according to a previously established protocol [1, 15, 36]. A mixture of 20 μl proteoliposomes and 280 μl of C13 buffer was introduced into the flow cell. Following pore insertion, a Platform Quality Check program was executed to monitor current changes. Baseline current was recorded at 100 mV before adding 300 μl of C13 buffer containing 50 nM substrate to the flow cell, after which current measurements were resumed at 100 mV. Data visualization was performed using custom software provided by Oxford Nanopore Technologies.

### Statistical analysis

Statistical analyses were performed using GraphPad Prism version 8. For comparisons involving more than two groups, one-way analysis of variance followed by Tukey’s post-hoc test was used. All experiments were confirmed a minimum of three times to ensure replicability. The sucrose gradient experiments were confirmed a minimum of three times each, but only one experiment is shown for ease of reading.

## Results

### Analysis of the sedimentation profile of procapsid using dsDNA, DNA/RNA hybrid, and single-stranded oligo fragments

**Figure 1.**
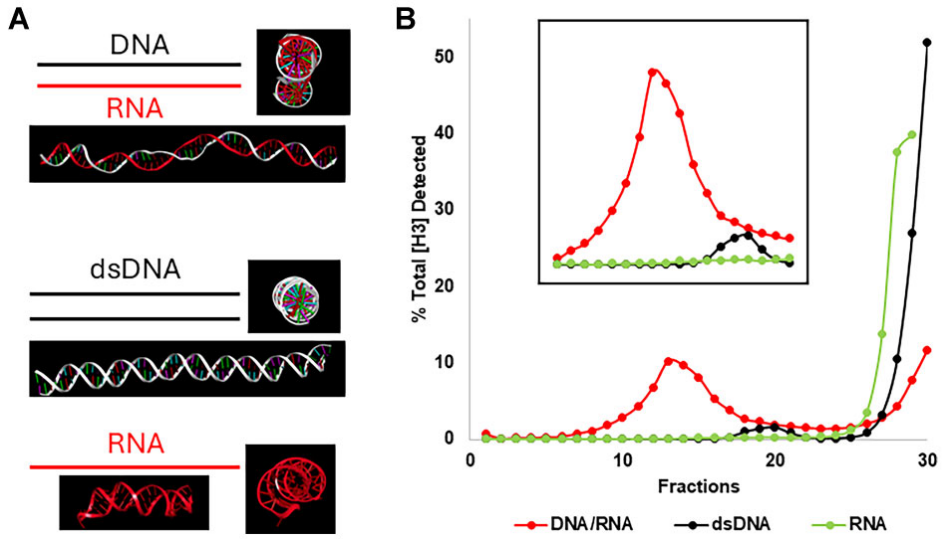
Packaging of RNA/DNA hybrid strands into the phi29 viral capsid at significantly higher amounts than dsDNA. (**A**) AlphaFold predicted the structure of the DNA/RNA hybrid strand (top), dsDNA strand (middle), and RNA strand (bottom), respectively, used for the packaging assay. (**B**) 5–20% sucrose gradient ultracentrifugation of the phi29 viral capsid packaged with the [^3^H]-labeled RNA/DNA, dsDNA, or RNA, respectively. The direction of sedimentation is from left to right. The [^3^H] signal of RNA/DNA hybrids migrated further into the left of the sucrose gradient, corresponding to heavier capsid encapsulated with a large copy number of [^3^H]-RNA/DNA hybrids compared to [^3^H]-dsDNA, whereas the [^3^H]-RNA alone did not sediment into the gradient at all.

In 5–20% sucrose gradient ultracentrifugation experiments of phi29, the sedimentation profile of procapsid packaging dsDNA, DNA/RNA hybrid, and single-stranded RNA (Fig. 1A and B, and Table 1) shows significant differences in sedimentation rate. The sedimentation rate is an indicator of the amount of materials in each complex. The more the DNA or RNA in the procapsid, the faster the procapsid moves into the gradient. The amount of total CPM represents the total amount of nucleotides in each fraction. The sizes of individual dsDNA and the DNA/RNA fragment were very similar, and the packaging efficiencies were similar under the same conditions; thus, the high CPM yield with faster migration rate of the DNA/RNA hybrid over dsDNA indicates a higher copy number of RNA/DNA hybrids in each procapsid compared to the dsDNA.

### Enhanced packaging efficiency of RNA/DNA hybrids compared to the dsDNA

Sucrose gradient ultracentrifugation assays quantified the extent of encapsidation (Fig. 1A and B). DNA packaging with RNA/DNA hybrids (Fig. 1A) displayed significantly higher sedimentation rate shown in the gradient, with peaks reaching up to 60% migration further into the gradient compared to those loaded with dsDNA (Fig. 1B). The peak representing the amount of labeled DNA/RNA hybrid in the procapsid is much higher and had a fast migration rate in the gradient compared to the dsDNA substrate. As analyzed in the previous section, this suggests a higher copy number of RNA/DNA hybrids in the procapsid. The RNA-only control samples did not exhibit any appreciable encapsidation, underscoring the necessity of a DNA component to engage the phi29 motor’s packaging mechanism. The necessity of a DNA component for motor engagement emphasizes the importance of sequence-specific interactions in the packaging process: only one strand of DNA is sufficient in initiation or translocation of the substrate, but two strands of DNA are needed for the termination, gating, and conformational change of the motor channel.

### Role of nucleic acid orientation in motor function

**Figure 2.**
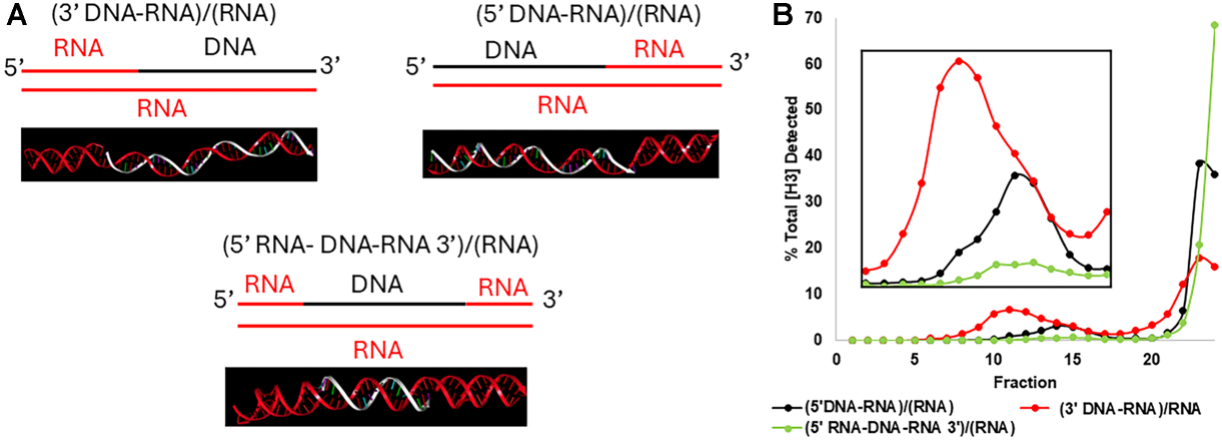
Testing of the terminal property for DNA or RNA strands for initiation on the packaging of the duplex. (**A**) AlphaFold predicted structures of DNA/RNA hybrid with DNA terminus at the 3′ end (top left), 5′ end (top right), and neither (bottom) used for the packaging assay with [^3^H]-UTP radioactive labeling on the conserved RNA strand. (**B**) 5–20% sucrose gradient ultracentrifugation of the phi29 capsid packaged with DNA terminus at different location, indicating that both 3′-DNA/5′-RNA hybrid and 5′-DNA/3′-RNA duplexes can be packaged.

**Figure 3.**
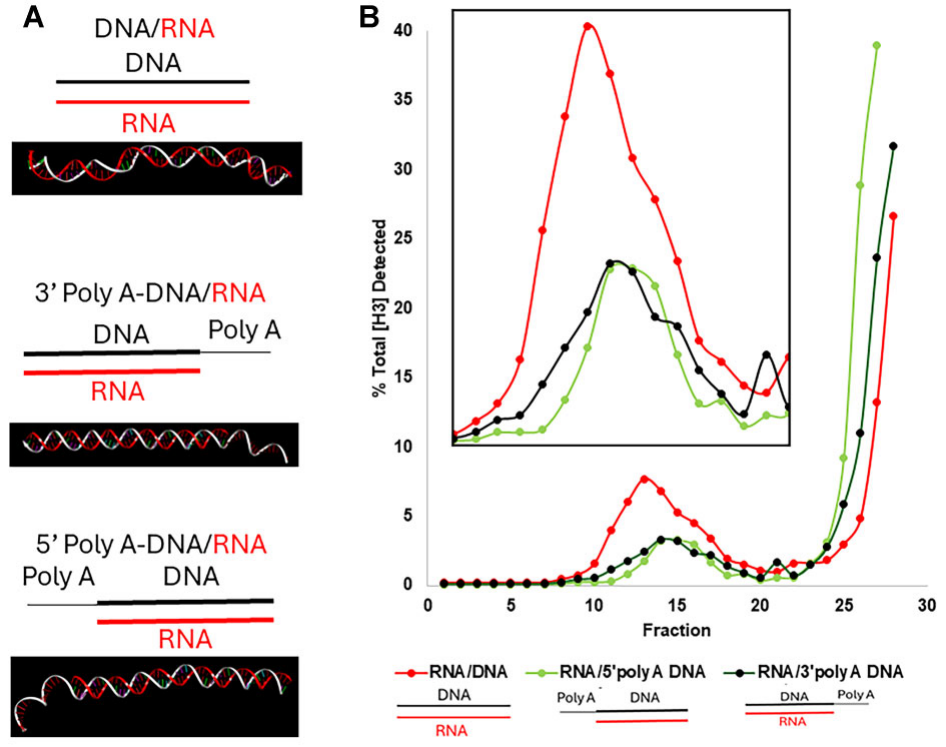
Further testing of the requirement of DNA terminus in DNA packaging. (**A**) AlphaFold predicted structures of the DNA/RNA hybrid: by itself (top), with 3′ polyA overhang (middle), and with 5′ polyA overhang (bottom) to be used for the viral packaging assay. (**B**) 20% sucrose gradient ultracentrifugation of the phi29 viral capsid packaged with materials in panel (A). The 3′ or 5′ polyA retarded packaging efficiency, due to the 50% reduction of the required ends with DNA.

To investigate the influence of nucleic acid orientation on the translocation efficiency of the phi29 motor, we examined the impact of 3′- and 5′-DNA/RNA presentation on packaging efficiency (Fig. 2A and B). RNA/DNA hybrids with either a 3′-DNA/5′-RNA or a 5′-DNA/3′-RNA can be packaged, supporting the conclusion in the previous two sections that emphasizes the importance of sequence-specific interactions in the packaging process, but only one strand of DNA is sufficient in the initiation or translocation of the substrate. The results revealed that RNA/DNA hybrids with a 3′-DNA/5′-RNA or a 5′-DNA/3′-RNA both can be packaged. Since it has been reported that the ATPase gp16 moves along the dsDNA from the 5′ to 3′ strand, we speculated that such a difference in findings regarding orientation and chirality is due to the data obtained between the studies on dsDNA translocation and DNA packaging initiation. There is no discrimination for the 3′-DNA/5′-RNA or 5′-DNA/3′-RNA in the initiation of DNA packaging. However, further clarification is needed since the packaging was analyzed by an ensemble signal but not a single-molecule study to distinguish the initiation and termination. Interestingly, the dsRNA that lacks DNA/RNA presentation on one end of the nucleotide structure resulted in much lower packaging efficiency, indicating that initiation is specified toward the end presenting DNA. Selective blocking of one of the DNA/RNA hybrid ends with 20-nt polyA on either side of the DNA 3′ or 5′ end, respectively, supports this role of at least one DNA terminus in the dsDNA strand for binding and initiation of the DNA packaging (Fig. 3A and B). The polyA was able to limit the procapsid packaging efficiency equally on either side of the hybrid duplex, indicating that the initiation was hampered by one of the DNA termini, either at the 5′ end or at the 3′ end, by the addition of the polyA regardless of orientation.

### DsDNA induced channel gating, whereas RNA/DNA hybrids did not

**Figure 4.**
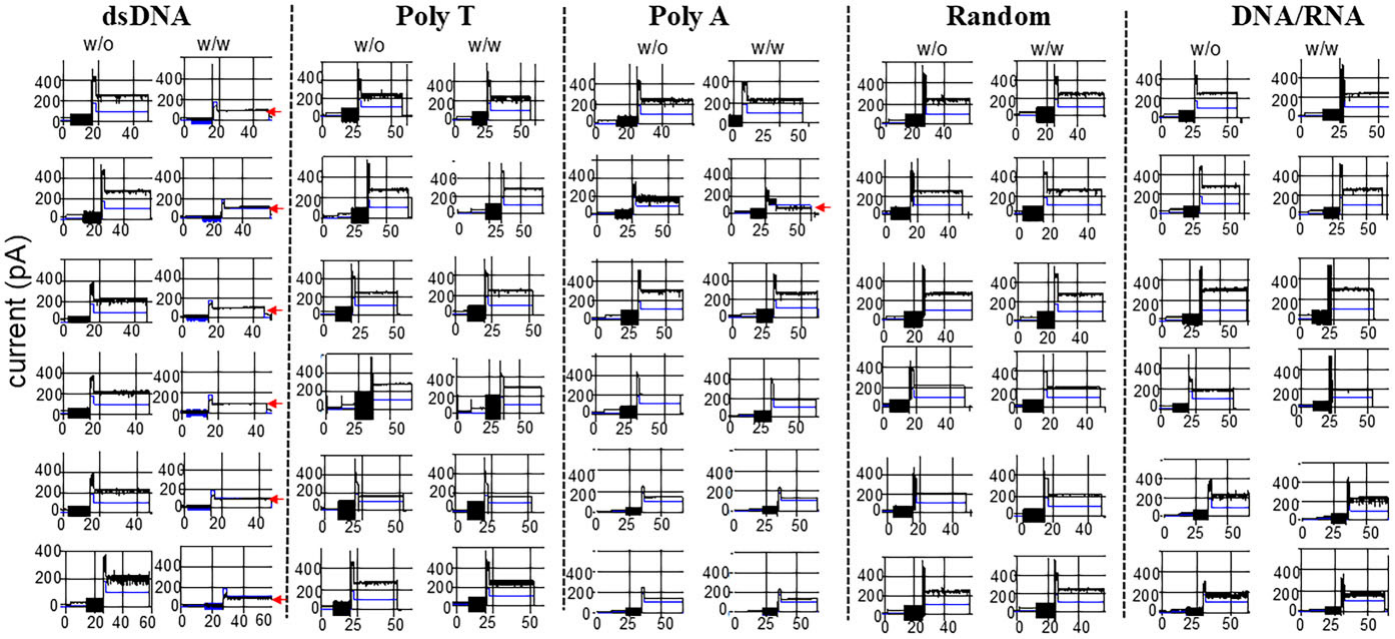
Current traces of the phi29 connector show a current reduction in response to gate closure during the translocation of dsDNA. Representative current changes without (left) or with (right) the addition of dsDNA, polyT, polyA, random sequence, or DNA/RNA. The arrow indicates the current reduction due to gating. Total channel numbers: dsDNA, 30; polyT, 20; polyA, 12; random, 8; DNA/RNA, 6.

**Figure 5.**
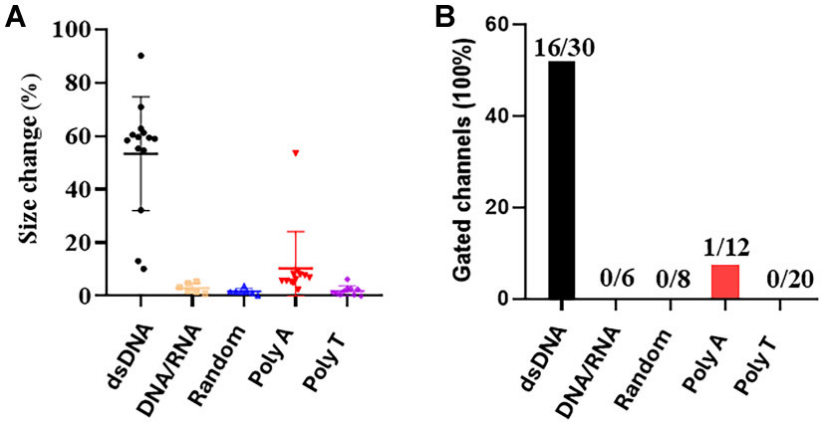
Column plot to assess the difference in gating caused by the DNA or RNA substrates. (**A**) The current reduction is due to gate closure during the translocation of dsDNA. Gating does not occur in the cases of DNA/RNA, as well as polyA and polyT strands. These results, indicate dsDNA specifically reduces the gate size of the channel. (**B**) Percent of total gated channels detected is a indication of a channel response to the translocation of the nucleic acid substrate.

To understand why RNA/DNA hybrid can be packaged with high copy number into the procapsid, we conducted single-channel electrophysiological assays comparing dsDNA and RNA/DNA hybrids with polyA, polyT, and a scrambled random sequence. Compared to lipid bilayer, the MinION™ flow cell polymeric membrane has shown itself to be more mechanically stable and resistant to high voltage. The advantages of number (2048 regions for pore insertion) and stability permit the MinION™ flow cell to be a promising platform for high-throughput analysis. As shown in Fig. 4, adding dsDNA but not RNA/DNA hybrids to the motor channels resulted in substantial gating events, characterized by pronounced reductions in ionic current, indicative of gate closure (Table 2). The addition of polyA or polyT sequence hardly induced channel gating; only 1 of 32 channel gating was observed (Fig. 5A). In contrast, 16 of 30 channels showed induced gating after adding dsDNA (Fig. 5B). The geometric mean of channel size reduction was 46.7, 2.5, 7.5, 0.9, and 1.4 after the addition of dsDNA, RNA/DNA hybrid, polyA, polyT, and random sequence, respectively (Fig. 5A). Significantly, the RNA/DNA hybrids did not cause gating, supporting the idea that high yield of the packaging process is a result of the lack of gating and termination during dsDNA packaging.

**Table 2.** Results of channel size reduction ratio

dsDNA		DNA/RNA		PolyA		PolyT		Random	
Channel no.	Channel size reduction ratio	Channel no.	Channel size reduction ratio	Channel no.	Channel size reduction ratio	Channel no.	Channel size reduction ratio	Channel no.	Channel size reduction ratio
83	90.095663	88	5.4385262	5	7.8003224	48	1.8036547	193	1.7208514
193	13.143007	144	4.8042787	55	5.3910102	55	0.5318659	212	0.2839563
342	10.216964	277	3.3224682	84	6.3742111	76	2.7281589	317	1.6540716
391	70.839027	323	0.8534892	136	53.543001	81	0.007382	346	3.8527238
88	61.231236	355	1.946913	212	2.3939236	82	2.7674394	399	1.583245
144	59.655698	428	1.5299985	346	9.2198984	113	2.5783378	398	1.6307697
277	55.321344			371	4.963322	122	0.465068	511	1.0946965
312	59.336333			398	5.7356239	0.81	0.8108905		
323	54.621813			414	8.1625803	193	6.281611		
355	62.782899			467	6.9257638	212	1.1211879		
360	58.377329			511	8.4091763				
428	32.22124								
455	60.467313								
512	58.968018								

*Note*: “Channel size reduction ratio” is calculated as{(the channel current without DNA or RNA)-(the channel current with DNA or RNA)} / (the channel current without DNA or RNA)×100%. ****P < .0001. The gating % calculation is as follows. The narrowest side of the phi29 connector channel is 3.6 nm, and the area of the channel is (3.6 nm/2)^2^ × 3.14 = 10.17 nm^2^. The area of the dsDNA is 2 nm, and (2 nm/2)^2^ × 3.14 = 3.14 nm^2^. 10.17 nm/3.14 = 32%.

## Discussion

### Mechanism of gating: interaction of dsDNA with the flexible loops

Previous studies have demonstrated the tendency of dsDNA to induce significant channel gating as long as the dsDNA touched one of the connector flexible loops, either the C-terminal of the connector protein gp10 or the internal loop that is located within the internal location of the connector channel, due to dsDNA’s structural rigidity and interactions with the positively charged lysine residues lining the phi29 motor channel [1, 3, 15, 36]. These electrostatic interactions between the basic amino acids and the negatively charged phosphate backbone of dsDNA create a constrained environment, impeding translocation efficiency. In stark contrast, RNA/DNA hybrids exhibited minimal gating effects, as reflected by the comparatively stable current traces. This indicates that RNA/DNA hybrids are able to maintain an open channel conformation during translocation. The observed differences suggest that RNA/DNA hybrids can be packaged with more than one copy without gating compared to dsDNA, potentially due to their A-form helical structure. This structure, characterized by a reduced base rise of ∼0.26 nm and a helical pitch of 2.86 nm [8], may facilitate smoother passage through the motor channel by reducing steric hindrance and weakening electrostatic interactions. Unlike the bulkier B-form structure of dsDNA, which exhibits a base rise of 0.34 nm and a pitch of 3.36 nm [33, 54], the looser helical configuration of RNA/DNA hybrids may allow for faster and more efficient translocation. Our findings indicate that the structural adaptability of the phi29 motor enables it to accommodate various nucleic acid substrates with differing helical properties. The reduced gating observed with RNA/DNA hybrids could also be attributed to their flexibility, which may prevent configuration interactions with the lysine residues.

Phi29 connector protein contains three flexible loops: the N-terminal loop amino acids (aa) 1–14, the internal loop aa 229–246, and the C-terminal loop aa 287–309. The structures of these three loops were not included in the crystal structure due to their flexible nature. To investigate whether these three loops are involved in channel gating, three connector mutations, with loop 1–14, 229–246, and 287–309 changes, have been constructed. The stepwise gating was observed. The results suggest that phi29 connector loops implemented a conformational change with three discrete steps. It was suggested that these three steps of conformational change are regulated by the interaction of DNA with the connector loop.

### RNA/DNA hybrids as preferred substrates: mechanistic insights into channel dynamics

The stark contrast in packaging efficiency between RNA/DNA hybrids and dsDNA, as highlighted by our single-channel electrophysiological assays (Fig. 5), challenges the traditional view that dsDNA is the optimal substrate for DNA bacteriophage packaging motors. The significantly reduced gating events observed with RNA/DNA hybrids suggest that the phi29 motor channel remains more open during translocation of these hybrids. This can be attributed to their A-form helical structure, characterized by a smaller base rise of ∼0.26 nm and a helical pitch of 2.86 nm [1, 22, 30–35, 54]. In contrast, the B-form structure of dsDNA, with a base rise of 0.34 nm and a pitch of 3.36 nm, results in stronger electrostatic interactions with the 48 positively charged lysine residues lining the phi29 motor channel [1, 22, 30–35, 54]. This creates substantial steric and electrostatic resistance during dsDNA translocation. The flexibility of RNA/DNA hybrids, facilitated by their A-form helix, likely leads to reduced steric hindrance and electrostatic resistance, allowing for smoother and faster translocation. This finding aligns with previous studies involving other ATP-driven motors, such as those in T4 and lambda phages, which exhibit higher packaging efficiency for substrates with increased flexibility and reduced helical rigidity [28, 37, 55–63, 65]. Our results indicate that the structural adaptability of the phi29 motor enables it to exploit the helical properties of RNA/DNA hybrids, making them more efficient substrates than the traditionally studied dsDNA.

### Orientation-dependent translocation: implications for efficiency in initiation of packaging

Sucrose gradient ultracentrifugation assays (Fig. 2) provided quantitative evidence of the enhanced encapsidation efficiency of RNA/DNA hybrids, which sedimented further into the gradient compared to dsDNA. This indicates a significantly higher extent of packaging for RNA/DNA hybrids, suggesting a structural adaptability of the phi29 motor to accommodate less rigid helical conformations. The RNA-only controls, which displayed negligible sedimentation, emphasize the essential role of a DNA component in engaging the motor for effective translocation. The preference for RNA/DNA hybrids over dsDNA may reflect an evolutionary adaptation by the phi29 motor to optimize packaging efficiency under conditions where substrate flexibility varies.

Our investigation into substrate orientation (Fig. 3) reveals that the phi29 motor can package RNA/DNA hybrids with a 3′-DNA/5′-RNA or a 5′-DNA/3′-RNA configuration. It has been reported from other viral systems, where the orientation of nucleic acids relative to the motor’s entry site significantly influences encapsidation efficiency [1, 57, 59, 65]. Given that RNA/DNA hybrids can be packaged at much higher rates than dsDNA, this could have profound implications for designing synthetic viral vectors to optimize cargo loading. It has been reported that this ATPase motor moves along the dsDNA from the 5′ to the 3′ strand [1, 66, 67], leading to the speculation that the chemical requirement is for the moving of the substrate but not for the initiation.

### The implication of structural flexibility of DNA/RNA hybrid strands in gating

Previously, it was reported that A-form nucleic acid was less rigid, which led to different translocation rate. In this report, we cannot conclude that the structure flexibility of RNA/DNA hybrid is inducing gating of the motor. It is possible that structural flexibility in DNA/RNA hybrids might play a role in regulating the gating efficiency of the phi29 motor channel [21, 41]. This flexibility of the DNA/RNA manifests in both bending and torsional flexibility. The bending flexibility reduces the cost for bending the strand. The torsional flexibility reduces the cost of handoff between gp16 subunits. How these properties of structure flexibility would affect the packaging events observed between DNA/RNA hybrids and dsDNA would be critical to interpret the mechanism of substrate and channel-loop interaction (Figs 4 and 5). In theory, the initial contact and initiation events between the substrate and the channel complex would be a function of the bending and torsion of the substrate in relation to the channel–substrate interaction [41, 68]. DNA/RNA hybrids, adopting an A-form conformation, might exhibit a narrower major groove and a shorter helical pitch compared to the B-form dsDNA, leading to reduced electrostatic interactions with the positively charged residues within the phi29 motor channel that does not induce the required electrostatic conditions for gating to occur upon recognition of the substrate (dsDNA, DNA/RNA hybrid) and channel [31, 35, 69]. These structural differences may contribute to the observed reduction in gating events due to difference in the electrostatic properties of the DNA/RNA that allow for more initiation of new packaging events.

The physiological relevance of these findings can extend beyond the immediate observations *in vitro*, particularly in the context of the viral genome triggering gating constraints *in vivo*. The physics of translocation for longer genomic substrates may introduce additional structural constraints, including helical overwinding and increased intracapsid pressure, which could exacerbate the gating effects observed for dsDNA [14, 21, 28, 29]. However, it is notable that the electrophysiology experiments conducted in this report with the isolated connector protein suggest that gating occurs independently of intracapsid steric pressure effects, reinforcing the idea that dsDNA translocation is terminated primarily by gating rather than by reaching a steric limit imposed by intracapsid crowding. The possibility that intracapsid pressure contributes to gating termination in dsDNA, but to a lesser extent in DNA/RNA hybrids, warrants further exploration as to why it does not seem to be related in our experimental results.

The potential impact of purine/pyrimidine (Pu/Py) asymmetry in shaping the structural properties of DNA/RNA hybrids for gating is another key consideration. While our study did not explicitly test Pu/Py asymmetry variations, previous studies suggest that sequence composition can significantly alter DNA/RNA hybrid rigidity and, as discussed earlier, the role in the physical properties of the substrate in triggering gating upon binding [69, 70]. Incorporating this factor into future investigations may help further elucidate the role of sequence-dependent structural differences in hybrid gating dynamics. The observed increase in packaged DNA/RNA hybrid molecules arises from their ability to bypass gating mechanisms.

### Future directions in application

These findings provide a structural and chemical basis for developing containers to package RNA/DNA hybrids for nucleic acid delivery, gene therapy, or single-particle sensing by packaging fluorescent or radiolabeled DNA or RNA oligos into the container. The container will serve as an unusually strong imaging marker. After inserting the connector from the motor into a specific device, it becomes possible to deliver dsDNA sequences coding for Cas9 for delivery, enabling the quantitative delivery of therapeutic dsDNA. Additionally, utilizing the same principle and equipment, it is also possible to achieve the large-scale loading of RNAi into cells’ 2′ modification at one of the dsRNA strands.

## Conclusion

RNA fragments cannot be packaged; dsDNA fragments can be packaged but lead to gating. RNA/DNA hybrids can be packaged with high copy numbers due to the lack of gating by the RNA/DNA hybrids. One DNA terminus, either at the 5′ end or at the 3′ end in the packaging substrates, is necessary for the motor to package it. This emphasizes the importance of the 2′-OH group for specific interactions in the packaging process: Two strands of DNA are required for the motor channel’s termination, gating, and conformational change. The interaction of the dsDNA with the flexible channel loop is the cause of gating. The ability to package RNA/DNA hybrids with a more significant copy number than dsDNA, combined with the dsDNA terminal requirement for gating and the gating mechanisms, underscores its potential for applications in gene/RNAi delivery, gene therapy, synthetic biology, nanotechnology, and high-sensitivity single-particle sensing. Further studies focusing on the structural dynamics of the phi29 motor could provide deeper insights into optimizing packaging systems for various nucleic acid substrates, paving the way for more advanced pharmaceutical and biotechnological applications.

## Supplementary Material

gkaf242_Supplemental_File

## Data Availability

Data supporting the findings of this study are available within the article and its supplementary materials or are available on request.
