# Supplementary material for: DNA/RNA hybrids avoid channel gating that leads to the continued packaging of numerous hybrids into the phi29 protein shell
Source: Nucleic Acids Res. 2025 Apr 7;53(6):gkaf242. doi: 10.1093/nar/gkaf242 (PMC11975285; doi:10.1093/nar/gkaf242)
Supplement: gkaf242_Supplemental_File [file gkaf242_supplemental_file.pdf]

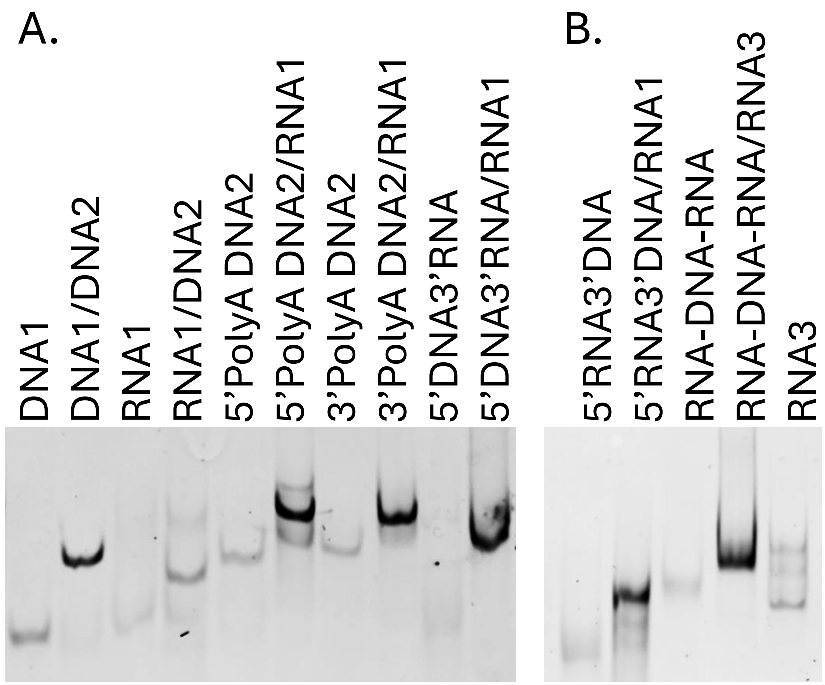

Supplemental Figure 1. Gel Electrophoresis of DNA/RNA Product. **A.** 12% TBE Native Polyacrylamide Gel. **B.** 8 % TBE Native Polyacrylamide Gel. For sequence specific composition look at table 1 in the methods section
